# Chaperones and the maturation of steroid hormone receptor complexes

**DOI:** 10.18632/oncotarget.238

**Published:** 2011-03-11

**Authors:** Yizhi Jane Tao, Wenjie Zheng

**Affiliations:** ^1^ Department of Biochemistry and Cell Biology, Rice University, 6100 Main Street, MS140, Houston, TX 77005, USA

Steroid hormone receptors (SHRs) of the nuclear receptor family are intracellular, ligand-dependent transcription factors responsible for sensing endogenous steroid hormones that diffuse through the cell membrane. Ligand binding to a SHR causes conformational changes in the receptor that enable it to directly bind to DNA (*i.e.* HREs - the hormone response elements) and regulate the expression of adjacent genes [1]. Due to the large number of genes that they regulate, SHRs play important roles in controlling the development, homeostasis, and metabolism of the organism. The malfunctioning of SHRs signaling pathways has also been implicated in the development of many forms of cancers.

SHRs have a conserved structure with at least four modular domains, a variable N-terminal transactivation domain, a DNA-binding domain (DBD), a short hinge region, and a hormone-binding domain (HBD) that also has the transactivation function [2]. The best studied steroid hormone receptors are members of the nuclear receptor subfamily 3, such as the androgen receptor, estrogen receptors, glucocorticoid receptor, and progesterone receptor [3-7]. Depending on their steroid hormone specificity, SHRs are either located in the cytosol and migrate to the cell nucleus upon activation (e.g. glucocorticoid receptor), or are predominantly nuclear in both unliganded and liganded forms (e.g. progesterone receptor) [8]. The nuclear import of receptors is mediated by the nuclear localization signals (NLSs), which have been identified in the DBD-hinge and the HBD regions of the SHRs [9-11]. In most cases, the NLSs are covered up by heat shock proteins which remain bound with the receptor until the hormone ligand is present (as discussed below).

After their synthesis, SHRs must go through a heat shock protein-assisted maturation process before they become receptive for ligand binding. During maturation, SHRs are first delivered by Hsp70 to Hsp90 through the Hsp organizing protein (Hop), which binds both chaperones through two separate tetratricopeptide repeat (TPR) domains [12]. TPR domains are a-helical solenoid structures consisting of three or more tandem tetratricopeptide repeats each containing a highly degenerate sequence of 34 amino acids [13]. Both Hsp90 and Hsp70 interact with co-chaperones containing TPR domains through a short C-terminal sequence -EEVD-. The Hsp90-SHR complex is further stabilized in its ATP-bound form by p23, a small acidic protein generally involved in the stabilization of client protein -Hsp90 complexes [14]. The presence of ATP weakens the affinity of Hsp90 for Hop, thus allowing for the recruitment of another TPR protein: FKBP52, FKBP51, Cyp40 or PP5. FKBP52 and FKBP51, and Cyp40 are also known as the large immunophilins because they contain a peptidyl prolyl isomerase domain that can bind immunosuppressive drugs [15]. Finally, this assembly and maturation process produces a large, oligomeric SHR-Hsp90 complex with high hormone binding affinity, as Hsp90 and co-chaperones maintain the SHR in a particular structural conformation that is highly responsive to hormone [16]. It is currently unclear whether hormone-bound SHRs translocate through the nuclear pores with or without the Hsp90 complex. Nevertheless, it is generally accepted that the Hsp90-SHR interaction is disrupted upon hormone binding [17].

To analyze the interactions among various chaperones and co-chaperones during the maturation of SHRs, Hildenbrand *et al* report in this issue an intriguing finding that Hsp90 can accommodate the simultaneous binding of the FKBP52 and HOP proteins, in striking contrast to previous findings that HOP and FKBP52 binding to Hsp90 is mutually exclusive [18-20]. Using highly purified recombinant proteins, Hildenbrand *et al* carried out detailed biochemical and biophysical characterization of the various complexes and subcomplexes that the four chaperones/co-chaperones, Hsp90, FKBP52, p23 and HOP, are able to form in the presence and absence of nucleotides. In particular, Hildenbrand *et al* identified a stable Hsp90_2_-FKBP52_1_-p23_2_-HOP_2_ complex with the 2:1:2:2 molar ratio. The simultaneous binding of FKBP52 and HOP the Hsp90 dimer is independent of nucleotide binding and the p23 co-chaperone. The authors speculate that Hsp90 may have two separate binding sites for FKBP52 and HOP, one at the C-terminal -EEVD- motif and the other at the hydrophobic middle-domain of Hsp90. Further structural investigations, such as electron microscopy as suggested by the authors, would be useful to elucidate the details of such intermolecular interactions. The possible existence of such a pre-assembled Hsp90_2_-FKBP52_1_-p23_2_-HOP_2_ complex ready for Hsp90-SHR binding in the cytosol raises an interesting possibility that the maturation pathway for SHRs may take fewer intermediate steps than we originally thought. Indeed, pre-assembled complexes are commonly found in cellular processes such as transcription (e.g. TFIID and mediator) and translation (e.g. eIF3). The results reported here thus constitute a giant step toward our understanding of the maturation process of SHRs.

Future experiments using purified SHRs are necessary to investigate how receptor binding may change the overall stoichiometry and structure of the chaperone/co-chaperone complex. It is unclear whether HOP and p23 remain associated in the mature Hsp90-SHR complex. One thing we can be sure of, however, is that the work by Hildenbrand et al have laid solid foundation for further biochemical and structural characterization of various hormone receptor-chaperone complexes at the molecular level.
